# Nanomaterial Nitric Oxide Delivery in Traumatic Orthopedic Regenerative Medicine

**DOI:** 10.3389/fbioe.2020.592008

**Published:** 2021-01-18

**Authors:** Albert Thomas Anastasio, Ariana Paniagua, Carrie Diamond, Harrison R. Ferlauto, Joseph S. Fernandez-Moure

**Affiliations:** ^1^Duke University Health System, Durham, NC, United States; ^2^Duke University School of Medicine, Durham, NC, United States

**Keywords:** nitric oxide, bone, bone healing, fracture repair, osteoinduction, biologic, biologic therapy

## Abstract

Achieving bone fracture union after trauma represents a major challenge for the orthopedic surgeon. Fracture non-healing has a multifactorial etiology and there are many risk factors for non-fusion. Environmental factors such as wound contamination, infection, and open fractures can contribute to non-healing, as can patient specific factors such as poor vascular status and improper immunologic response to fracture. Nitric oxide (NO) is a small, neutral, hydrophobic, highly reactive free radical that can diffuse across local cell membranes and exert paracrine functions in the vascular wall. This molecule plays a role in many biologic pathways, and participates in wound healing through decontamination, mediating inflammation, angiogenesis, and tissue remodeling. Additionally, NO is thought to play a role in fighting wound infection by mitigating growth of both Gram negative and Gram positive pathogens. Herein, we discuss recent developments in NO delivery mechanisms and potential implications for patients with bone fractures. NO donors are functional groups that store and release NO, independent of the enzymatic actions of NOS. Donor molecules include organic nitrates/nitrites, metal-NO complexes, and low molecular weight NO donors such as NONOates. Numerous advancements have also been made in developing mechanisms for localized nanomaterial delivery of nitric oxide to bone. NO-releasing aerogels, sol- gel derived nanomaterials, dendrimers, NO-releasing micelles, and core cross linked star (CCS) polymers are all discussed as potential avenues of NO delivery to bone. As a further target for improved fracture healing, 3d bone scaffolds have been developed to include potential for nanoparticulated NO release. These advancements are discussed in detail, and their potential therapeutic advantages are explored. This review aims to provide valuable insight for translational researchers who wish to improve the armamentarium of the feature trauma surgeon through use of NO mediated augmentation of bone healing.

## Introduction

An estimated 7.9 to 15 million fractures are sustained annually in the United States (Bishop and Einhorn, 2007; Bigham-Sadegh and Oryan, 2015). Fractures may result from trauma, osteoporosis, overuse, tumors, or genetic factors, and contribute to increased mortality and reduced quality of life (Bigham-Sadegh and Oryan, 2015). The delayed-union and non-union rate is 5–20% in the overall population, however, in the presence of vascular injuries it increases to almost 50% (Hu et al., 2017). Patients with non-union have higher rates of all-type healthcare utilization, undergo more surgical procedures, and are more likely to use high doses of strong opiates for pain control (Antonova et al., 2013). In patients with tibial shaft non-union, the median cost of total care is $25,556, two times more than in patients with normal fracture healing (Antonova et al., 2013). The costly socioeconomic and personal burden of fractures, especially non-healing fractures, has led investigators to study the underlying mechanisms in order to provide a solution to this complex problem.

Fracture non-healing has a multifactorial etiology and many risk factors. Environmental factors such as wound contamination, infection, and open fractures can contribute to non-healing (Bigham-Sadegh and Oryan, 2015). Patient-related factors such as smoking, diabetes, rheumatoid arthritis, immunodeficiency, or an immunocompromised state cause alterations in cytokine expression, which affects osteoclast activity and bone remodeling and prolongs fracture healing (Pape et al., 2002; Castillo et al., 2005; Kayal et al., 2007; Claes et al., 2012; Jeffcoach et al., 2014; Schneider et al., 2018). Lastly, sequelae of trauma such as shock and sepsis can impair fracture healing through the complex interplay of the immune system and regenerative response of the body to injury. While all these factors may inhibit the proper healing of bone, the course of fracture healing is largely influenced by stability via fracture site fixation and blood supply to the site of healing (Claes et al., 2012).

The current standard of care for fractures is urgent stabilization (Claes et al., 2012). Early definitive fixation and optimization of mechanobiology are crucial to fracture healing, as excessive instability at the fracture site can cause non-union (Schneider et al., 2018). There are many current methods of stabilizing fractures. Non-surgical approaches involve closed reduction of the fracture and external splinting with a cast or brace and is often used in pediatric fractures (Lien, 2017). In the case of concurrent open soft tissue wound or infection, provisional fixation can be achieved with external fixators or frames supporting percutaneously-pinned fracture fragments (Claes et al., 2012). Internal fixation of fractures can be accomplished with plates and intramedullary nails. Other guidelines for achieving union include minimizing local soft tissue injury, infection control, and avoidance of fracture hematoma debridement (Dimitriou et al., 2005; Metsemakers et al., 2018; Schneider et al., 2018).

## Process of Normal Bone Healing

Bone can heal by two mechanisms: primary healing (intramembranous ossification), which involves the deposition of bone by osteoblasts formed directly from mesenchymal stem cells, or secondary healing (endochondral ossification) which involves bone formation from a cartilage intermediate. Long bone fractures may heal by a combination of these two mechanisms, but the majority of fractures undergo repair by secondary healing (Bahney et al., 2019). Motion at the fracture site promotes repair through endochondral ossification, while stability promotes intramembranous ossification (Bigham-Sadegh and Oryan, 2015). Secondary healing of fractured bone has classically been defined as occurring in three stages—acute inflammation, repair, and remodeling—though the stages of healing are partially overlapping. The process of bone healing is complex and involves a delicate balance of many signaling and effector molecules.

**Figure 1:**
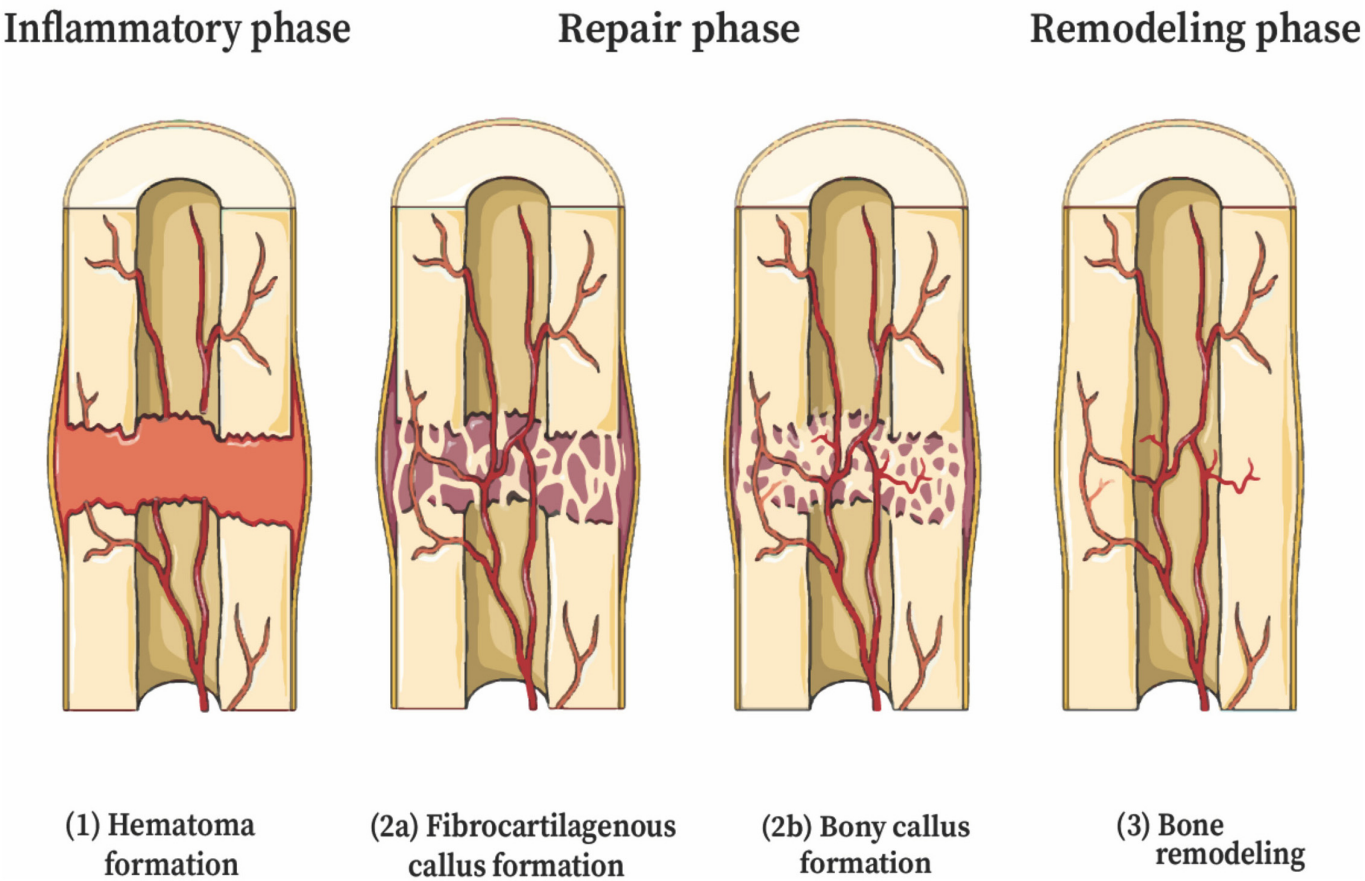
Stages of secondary bone healing (endochondral ossification). The stages of fracture healing are partially overlapping. (1) Coagulation and acute inflammatory response—initial stabilization, recruitment of inflammatory cells and cytokines. (2) Repair—(a) Revascularization, soft cartilage callus. Chondroblasts derived from MSCs deposit cartilage, mature to chondrocytes that express VEGF, inducing neovascularization; (b) Hard bony callus—hyperproliferative chondrocytes transdifferentiate to osteoblasts and osteocytes, tissue invaded by osteoblasts; (3) Remodeling—deposition of lamellar bone and restoration of pre-injury anatomic dimensions. This figure was created using Servier Medical Art templates, which are licensed under a Creative Commons Attribution 3.0 Unported License (https://creativecommons.org/licenses/by-sa/3.0/legalcode); https://smart.servier.com.

**Figure 2:**
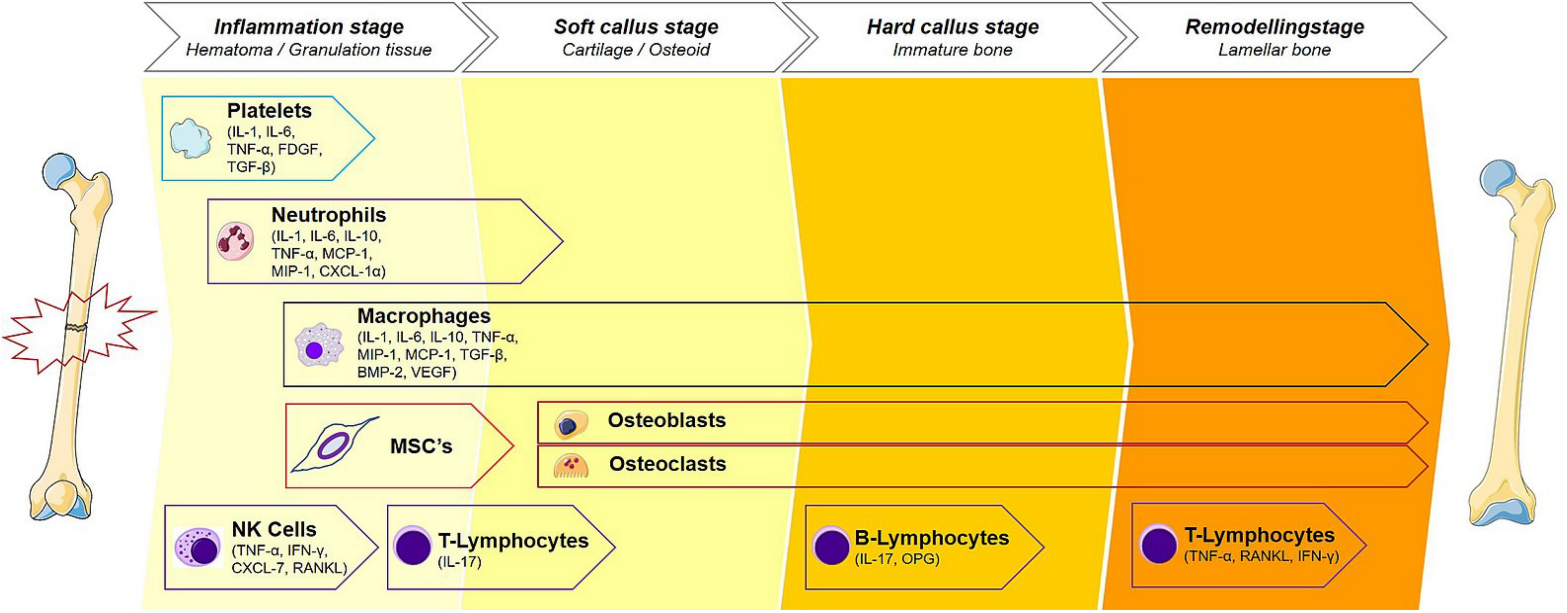
Immune cells of the fracture healing cascade for each stage of bone healing. The cellular contributions to fracture healing are described. Starting with platelets, a cascade of events that transition the bone from an inflammatory period to a proliferative period noted by soft and hard callus formation and predominated by reparative cells such as osteoblasts and osteoclasts. This phase then transitions to the remodeling phase where the healing site is structurally reinforced. Macrophage, being present throughout the healing cascade, release NO and play an important role throughout all stages of repair. Each stage of bone healing has a different spectrum of immune cells present at the fracture site. Reproduced with permission. This illustration was adapted by Martijn Hofman [licensed under Creative Commons CC-BY-SA 4.0] using the original illustration from Baht et al. (2018) [licensed under Creative commons CC-BY 4.0 (http://creativecommons.org/licenses/by/4.0/)] and images from Smart Servier Medical Art [licensed under Creative Commons CC-BY-SA 3.0 (https://smart.servier.com/)].

The first stage of fracture healing begins with coagulation and an acute inflammatory response. A hematoma forms at the fracture site, creating a fibrin-rich scaffold that serves as the initial matrix for healing. Disruption of blood supply from the periosteum and alteration in bone architecture trigger a response from inflammatory cells and cytokines. Cytokines recruit inflammatory cells to the hematoma, and these cells, particularly neutrophils and macrophages, help debride the injury and produce cytokines that influence healing (Bahney et al., 2019). Osteoclasts resorb fragments of necrotic bone at the fracture edge (Takeyama et al., 2014). The second stage is the repair phase, and begins with revascularization, starting at the periosteum and progressing toward the hematoma (Claes et al., 2012) (Figure 1). Immobilization of the fracture early in the repair stage is important for the proper formation of blood vessels. The hematoma progress to become granulation tissue, followed by a soft cartilaginous callus (Bigham-Sadegh and Oryan, 2015). Multipotent, mesenchymal stem cells (MSCs) derived from local periosteum, endosteum, and bone marrow are then recruited to the fracture site and begin forming a fibrovascular callus (Figure 2). Chondroblasts derived from periosteal MSCs make new, avascular cartilage that spans the fracture gap (Colnot, 2009). The cells then mature into hyperproliferative chondrocytes that express VEGF, inducing neovascularization of the cartilage (Bahney et al., 2019). The hyperproliferative chondrocytes transdifferentiate to osteoblasts and osteocytes, leading to bone formation (Zhou et al., 2014; Hu et al., 2017). Other chondrocytes undergo apoptosis and the tissue is invaded by osteoblasts (Maes et al., 2010; Dirckx et al., 2013). Osteoblasts lay down layers of osteoid, which then becomes calcified in an alkaline environment to form mineralized bone. Osteoblasts secrete receptor of activated nuclear factor kappa-B ligand (RANKL), which binds the RANK receptor on osteoclasts and induces osteoclast maturation and activation, thereby promoting a controlled level of simultaneous bone resorption that is core to the remodeling cycle of bone (Kalyanaraman et al., 2018). Endothelial cells also promote bone formation through bone morphogenic protein (BMP) (Bahney et al., 2019). Among other molecules, M2 macrophages mediate endochondral ossification (Schlundt et al., 2018), and matrix metalloproteinases (MMPs) degrade the extracellular matrix, allowing vascular invasion into the newly generated bone (Ding et al., 2018). Union is achieved at the end of the repair phase. The final stage of fracture healing is the remodeling stage, which may last 6–9 years in humans and accounts for 70% of fracture healing time (Bigham-Sadegh and Oryan, 2015). It is during this phase that cortical and cancellous bone are differentiated and the structural framework of the healed bone begins to take shape. Successful remodeling results in a bone with pre-injury anatomical dimensions. In reality, endochondral ossification and bone remodeling occur concurrently (Schindeler et al., 2008). Osteoclasts and osteoblasts work in concert to degrade immature woven bone and replace it with mature lamellar bone.

Several agents are options for the enhancement of normal bone healing. In patients with critical sized wounds, or irregular gaps in bone, space filling methods are used. Autograft, involving harvest of bone from one site and transfer to another site in the same patient, is considered the gold standard for bone grafting, though allograft bone can be used as well (Miller and Chiodo, 2016). Demineralized bone matrix, autologous bone marrow aspirates and synthetic bone graft substitutes are other options for bone replacement (Finkemeier, 2002). Synthetic grafts may be used with growth-promoting factors such as BMP, which plays a role in regulating bone deposition. BMP safety and efficacy in attaining fracture healing is equivalent to autograft and reduces intra-operative bone loss, suggesting it might be a better choice than autograft (Bishop and Einhorn, 2007; Chen et al., 2020). BMP has also been used in tissue engineering and can be used for custom flap construction (Bishop and Einhorn, 2007). Local delivery of other modulators of bone healing, such as nitric oxide, represents an area of growing therapeutic potential.

## Nitric Oxide in Wound and Bone Healing

Nitric oxide (NO) is a small, neutral, hydrophobic, highly reactive, gaseous free radical that can diffuse across local cell membranes and exert paracrine functions on neighboring cells. Its signals are primarily executed by the secondary messenger, cGMP (Pacher et al., 2007; Abaffy et al., 2019), and it acts in local tissue as a result of its limited half-life and short diffusion distance (Radi, 2018). NO is created from L-arginine via a reaction catalyzed by nitric oxide synthase (NOS), of which there are three isoforms: neuronal (nNOS, NOS1), inducible (iNOS, NOS2), and endothelial (eNOS, NOS3). The NOS isoforms thought to be most relevant to wound and bone healing are eNOS and iNOS, however all three isoforms play a role (van't Hof and Ralston, 2001). Although constitutively expressed at low levels, eNOS expression can be increased by means of cytokine signaling (via the IP3/akt pathway), exposure to estrogen, and increased shear stress on cells (van't Hof and Ralston, 2001). iNOS on the other hand is not typically expressed by cells, but is transcriptionally upregulated over a period of hours when exposed to pro-inflammatory cytokines such as interleukin-1 (IL-1) and tumor necrosis factor alfa (TNFα) (van't Hof and Ralston, 2001). Another potential source of NO in tissues includes the reduction of nitrite, a storage form of NO, to NO by xanthine oxidase (Li et al., 2008).

NO plays a role in many biologic pathways, most notably in inducing relaxation of vascular smooth muscle, and it also participates in wound healing through decontamination, mediating inflammation, angiogenesis, and tissue remodeling (Chae et al., 1997; Schulz and Stechmiller, 2006; Abaffy et al., 2019). Additionally, NO is thought to play a role in fighting wound infection by killing Gram-positive and Gram-negative organisms (Chouake et al., 2012). The mechanism by which NO kills pathogens is through the formation of reactive nitrogen oxide species (RNOS), which damage DNA, inhibit DNA repair, damage the cell wall, and increase production of genotoxic agents such as hydrogen peroxide (Schairer et al., 2012). The balance of NO concentration is crucial in wound healing, as it exhibits dose-dependent effects on its targets, with both under and overproduction of NO impairing wound healing (Schulz and Stechmiller, 2006). Overproduction leads to reaction of NO with superoxide to form the reactive RNOS, peroxynitrite (Pacher et al., 2007), which causes post translational modifications and cytotoxicity, especially in smokers and diabetics (Radi, 2018). Underproduction of NO is also detrimental to wound healing, and is implicated in decreased collagen synthesis (Schaffer et al., 1997). While some evidence suggests iNOS is not required for cutaneous wound healing (Bell et al., 2007), other data suggests production of NO via iNOS is important for type I and type III collagen deposition and thus increased wound strength (Shi et al., 2000; Moretti et al., 2012). In addition to having a significant role in cutaneous wound healing, NO also plays an important role in bone healing.

**Figure 3:**
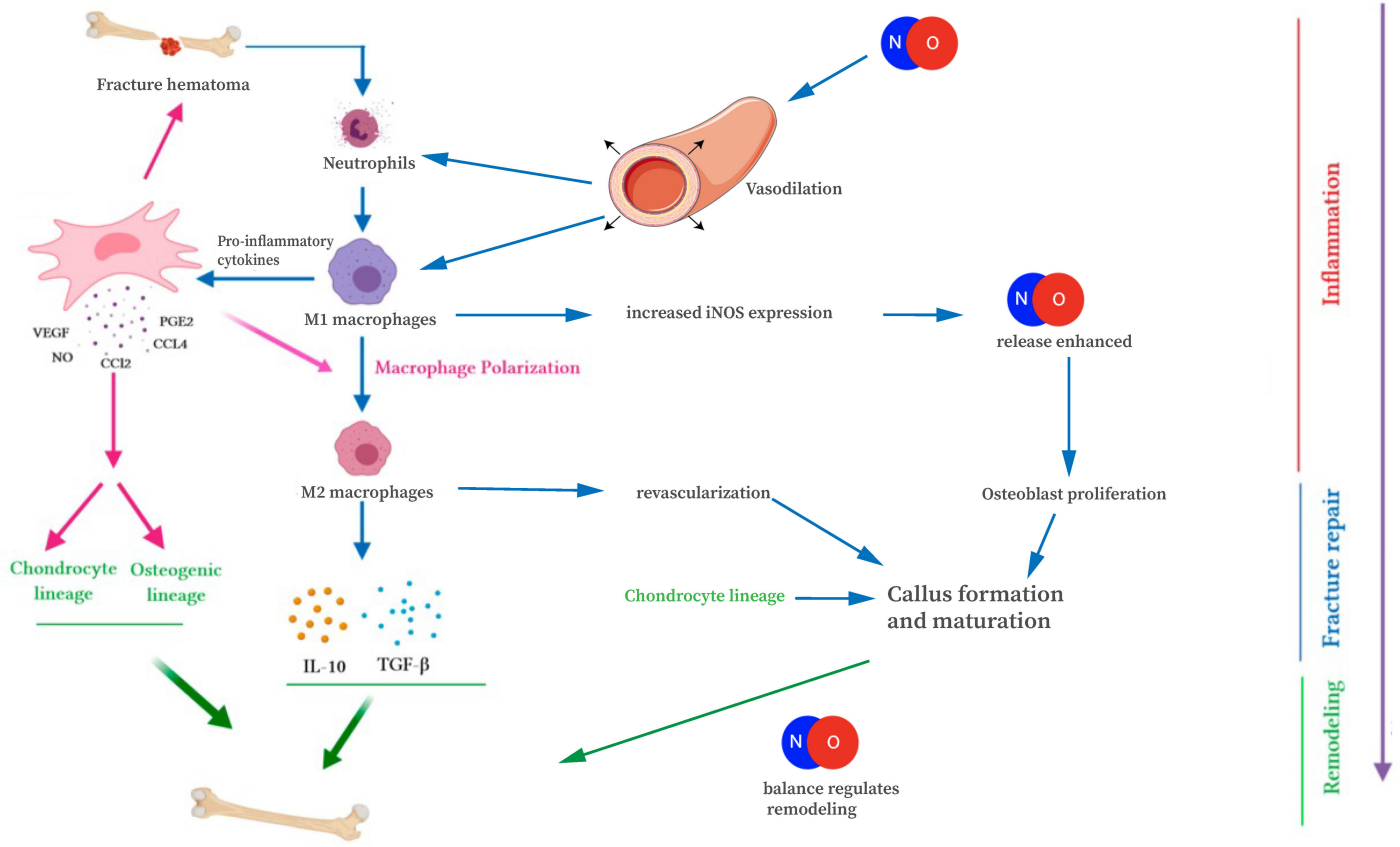
Interaction of NO with the immune cells of fracture repair. NO is involved in fracture repair through increasing blood vessel diameter, attracting immune cells, and mediating osteoblast/osteoclast differentiation and function. This figure was adapted using the original illustration from Medhat et al. (2019). [licensed under Creative commons CC-BY 4.0 (http://creativecommons.org/licenses/by/4.0/)] and images by Servier Medical Art templates, which are licensed under a Creative Commons Attribution 3.0 Unported License (https://creativecommons.org/licenses/by-sa/3.0/legalcode); https://smart.servier.com.

In bone healing, all three NOS isoforms are active during fracture repair, in a location and time-dependent manner. Cytokines, particularly IL-1 and TNFα, stimulate NO production through upregulation of iNOS and eNOS (van't Hof and Ralston, 2001). During the acute inflammatory phase of fracture repair, increased eNOS activity in the vasculature surrounding the fracture results in increased NO production, vasodilation, and facilitated delivery of inflammatory cells and cytokines to the fracture site (Tomlinson et al., 1985; Corbett et al., 1999). Importantly, patients with impaired vascular function exhibit delayed healing of stress fractures (Tomlinson et al., 1985; Ding et al., 2018). Neutrophils, macrophages, and mast cells migrate into the fracture site as part of the acute inflammatory response. Infiltrating macrophages and mast cells have increased iNOS expression, further enhancing NO release (Tomlinson et al., 1985; Chae et al., 1997; Zhu et al., 2001). The importance of NO-mediated attraction of inflammatory cells is evident later in bone healing as well, when M1 macrophages polarize toward M2 macrophages during endochondral ossification (Figure 3). Disturbances in this process may cause prolonged inflammation, delayed cartilage resorption, and impaired ossification leading to nonunion (Bastian et al., 2011; Schlundt et al., 2018). At the fracture site, levels of iNOS and eNOS increase within osteoblasts and periosteal cells, within a few days after injury (Corbett et al., 1999; Diwan et al., 2000; Zhu et al., 2001, 2002). Deficiency of these enzymes has been shown to result in a disturbed inflammatory response, prolonged neutrophil influx, and impaired healing and nonunion (Diwan et al., 2000; Meesters et al., 2016). By contrast, upregulation of the iNOS pathway has been shown to increase callus cross-sectional area and enhancement of early fracture healing (Diwan et al., 2000; Rajfer et al., 2017). iNOS and eNOS appear to play slightly different roles in callus formation, as evidenced by different callus volumes in knockout mice (Meesters et al., 2016). Thus, we begin to see the importance NO plays during the healing cascade.

**Figure 4:**
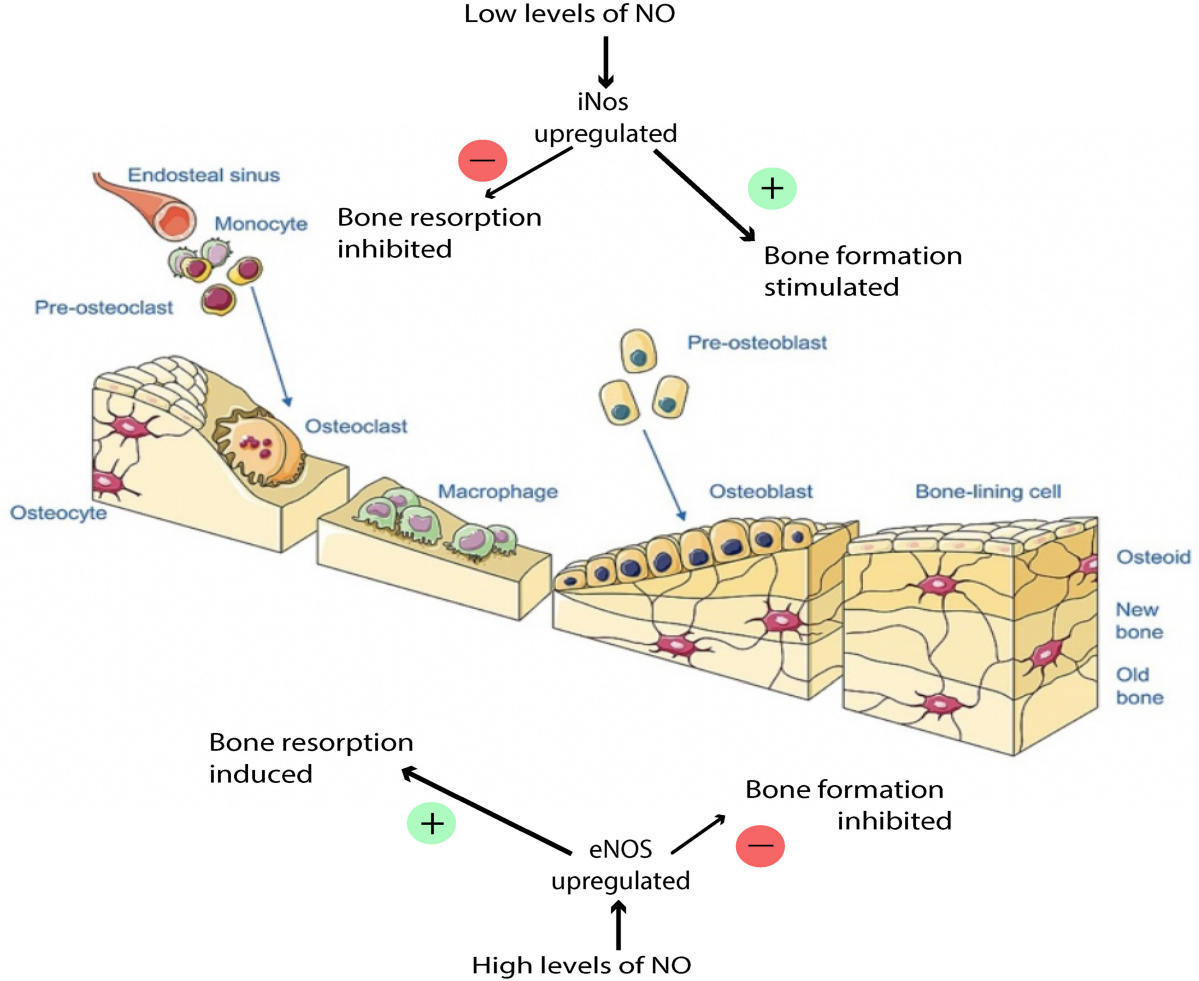
Effects of NO balance on bone remodeling. Low NO concentration stimulates osteoblast proliferation and bone formation. High NO concentration induces osteoblast apoptosis. This figure was created using Servier Medical Art templates, which are licensed under a Creative Commons Attribution 3.0 Unported License (https://creativecommons.org/licenses/by-sa/3.0/legalcode); https://smart.servier.com.

NO has a dose-dependent, biphasic effect on the activity of osteoblasts and osteoclasts (van't Hof and Ralston, 2001; Kalyanaraman et al., 2018) (Figure 4). Thus, during fracture repair, the balance of NO levels can be crucial to the proper execution of ossification and remodeling. The specific mechanism by which NO regulates osteoblasts and osteoclasts is complex and has not been entirely characterized, but has been shown to involve both cGMP-dependent and cGMP-independent pathways. In cells of the osteoblastic lineage, low doses of NO promote cellular differentiation, proliferation, and survival, and these effects are mediated by the cGMP pathway. In osteoblasts, the presence of NO activates soluble guanylate cyclase, leading to increased levels of cGMP, which has multiple downstream targets, such as cGMP-dependent protein kinases, which mediate bone growth (Teixeira et al., 2008; Kalyanaraman et al., 2018). NO has been shown to be constitutively produced at low levels in osteoblasts and is thought to protect osteoblasts from oxidative stress and help maintain skeletal homeostasis (Chang et al., 2006). The mechanism by which NO protects osteoblasts from oxidative stress is thought to involve an antiapoptotic mitochondrial pathway, and emerging evidence suggests that this pathway may specifically involve suppression of caspase activity through NO-mediated S-nitrosylation of caspases (Sun et al., 2006), however this is an area that warrants further investigation because it has not been fully elucidated. Additionally, NO released by osteoblasts mediates bone remodeling and vascular reactivity (Ding et al., 2018), and stimulates production of the precursors to collagen synthesis (Meesters et al., 2016). Mechanical loading can activate osteocytes to produce NO in response to fluid shear stress, transducing signals to regulate bone deposition and resorption (Klein-Nulend et al., 2014). While low NO concentrations stimulate osteoblast proliferation and bone formation, too high of an NO concentration induces osteoblast apoptosis through mechanisms that are still under investigation (Klein-Nulend et al., 2014; Abnosi and Pari, 2019). The specific concentration of NO in a localized sphere of influence is dependent on NO release from the donor species, but NO donor concentration of roughly 10 μM would be considered “low dosing” and has been linked to enhanced osteoblast function while 100 μM may be considered “high dosing” and has been linked to osteoblastic inhibition (Mancini et al., 2000).

In osteoclasts, low doses of NO are thought to also be necessary for proper osteoclast function (van't Hof and Ralston, 2001; Kalyanaraman et al., 2018). However, at high concentrations, NO inhibits osteoclast function through a cGMP-independent mechanism (Chae et al., 1997; Kalyanaraman et al., 2018). Osteoclast inhibition is detrimental to bone formation because without osteoclasts functioning to resorb bone in a controlled manner, bone remodeling cannot occur properly. Under pathological conditions, inflammatory cytokines such as IL-1 may over-induce the production of NO in bone cells via iNOS and thus disrupt bone tissue regulation (Lowik et al., 1994; Chae et al., 1997). NO also plays an inhibitory role when overproduced. Corbett et al. speculated that iNOS could be upregulated in conditions such as infection, causing alterations to the normal healing pathway by the production of high output NO, leading to free radical formation and the suppression of osteoclast activity (Corbett et al., 1999). Oxygen and nitrogen radicals while, antibacterial, may also play a role in affecting the cell membranes and DNA of regenerative cells locally. This inhibitory effect can stunt the wound healing cascade, and in the presence of infection, not allow progression of the wound healing cascade past the inflammatory phase. Localized delivery and tailored nanobiomaterials for the delivery of therapeutics must take into the consideration this inhibitory role.

Lastly, the antibacterial properties of NO in bone are also important. For example, it has been shown that NO-coated external fixation pins can reduce the risk of pin-site infection (Holt et al., 2011). This biomimetic approach leverages the natural processes by which nitrogen and oxygen radical formation in neutrophils cause cell membrane and DNA damage leading to bacterial cytotoxicity. Furthermore, NO exhibits an inherent broad spectrum of antimicrobial activity, and has been found to be effective against a wide array of bacteria (Schairer et al., 2012). Given the variety of beneficial roles NO plays in bone healing, investigators have sought modalities, such as nanomaterials, to localize and control delivery of NO to bone in order to enhance all stages of fracture repair.

The potential for NO-mediated enhancement of bone healing has led to the development of various exogenous delivery mechanisms. Aside from endogenous production, NO can also be created non-enzymatically from exogenous sources of NO, such as nitroglycerine. In this review, we will focus on localized NO delivery to bone, as well as to soft tissue injuries and infections that hold clinical relevance to fracture non-healing. Broadly categorized, currently investigated NO delivery types fall into two subgroupings: (1) NO donor molecules and (2) NO donor nanomaterials (Nichols et al., 2012) (Table 1).

**Table 1:** NO donors and delivery vehicles.

**Authors (PMID)**	**NO donor**	**Delivery vehicle**	**NO concentration**	**NO release half-life or duration**	***In vitro*/ *in vivo***	**Model system**	**Aim**	**Outcome**
Zhu et al. (2007)	Sodium nitrite	Gel	14.6 mM sodium nitrite mixed in equal amounts with maleic acid 14.6 mM + ascorbic acid 14.6 mM cream	Concentration of NO maintained at 10 mM for >1 h after application.	*In vivo*	Rats	Topical Gel-based NO donor effect on wound healing	Increased anti-inflammatory cell infiltration
Zhu et al. (2008)	Sodium nitrite	Gel	14.6 mM sodium nitrite mixed in equal amounts with low pH acid gel	Concentration of NO maintained at 10 mM for >1 h after application.	*In vivo*	Mice	Topical NO gel on wound healing	Increased re-epithelization by 50%, hair follicle regeneration, angiogenesis, procollagen—expressing fibroblasts, promotion and infiltration of inflammatory cells in wound beds
Phillips et al. (2004)	Sodium nitrite	Cream	6% wt/wt sodium nitrite mixed with 9.9% wt/wt citric acid cream	Not reported	*In vivo*	Human clinical trial	Topical nitrite cream effect on ulcer reduction	Reduction in surface area ulcers in Mycobacterium ulcerans wounds
Ormerod et al. (2011)	Sodium nitrite	Cream	3% (w/v) sodium nitrite mixed in equal amounts with 4.5% (w/v) citric acid in aqueous cream	Not reported	*In vivo*	Human clinical trial	Topical NO cream effect on MRSA wound clearance	Acidified topical nitrites able to clear 60% MRSA wounds
Martinez et al. (2009)	Topical NO	Chitosan derived hydrogel/ glass composite	100 nM peak, 50 nM steady state	Steady state reached after 6 h and maintained for 9 h, ongoing release occurred for ~24 h	*In vivo*	Mice	NO nanoparticle activity against MRSA wounds	Decreased eschar size, decreased bacterial burden, prevention of collagen degradation
Mihu et al. (2010)	Topical NO	Chitosan derived hydrogel/ glass composite	2.5 mg/ml of NO-np: initial peak 37.5 nM, steady state 20 nM 5 mg/ml of NO-np: initial peak 75 nM, steady state 50 nM 10 mg/ml of NO-np: initial peak 150 nM, steady state 100 nM 20 mg/ml of NO-np: initial peak 300 nM, steady state 200 nM	Not measured, reported in prior studies as ongoing release for ~24 h (Friedman et al., 2008; Martinez et al., 2009)	*In vivo*	Mice	NO-np activity on murine *Acinetobacter baumannii* wound model	Reduced suppurative infection, decreased microbial burden, reduced collagen degradation
Mancini et al. (2000)	SNP and 2,2' (hydroxynitrosohydrazino) bis-ethanamine (NOC-18)		SNP: 10μM, 50μM, 100μM NOC-18: 10μM, 50μM, 100μM	Not reported	*In vitro*	Rat enriched osteoblast cultures	NO effect on osteoblast activity	Slow, moderate NO release with NOC-18 stimulated osteoblast replication and alkaline phosphatase activity. Rapid high-concentration NO release with SNP inhibited proliferation and induced apoptosis
Abnosi and Pari (2019)	SNP		SNP 100 μM and 1,000 μM	Not reported	*In vivo*	Rats	Demonstrate possible effect of SNP as an NO-releasing agent on MSC differentiation to osteoblasts	SNP increased matrix deposition, promoted MSC differentiation to osteoblasts and may be useful in promotion of bone repair
Chen et al. (2019)	Dinitrosyl iron complexes (DNIC-1 and DNIC-2)	Direct application of DNICs into wounds	Angiogenesis: 7.8 μM DNIC-1, DNIC-2 Diabetic hindlimb ischemia: 0.18 mg/kg DNIC-	DNIC-1 *t*_1/2_ = 27.4 ± 0.5 h at 25°C and 16.8 ± 1.8 h at 37°CDNIC-2 *t*_1/2_ = 1.7 ± 0.1 h at 25°C and 0.8 ± 0.1 h at 37°C	*In vivo*	Mice	Effect of DNICs on wound healing	DNIC-1 displays best pro-angiogenesis and restores impaired angiogenesis in ischemic hind limbs, increasing wound repair in diabetic mice
Shekhter et al. (2015)	Dinitrosyl iron complexes with glutathione (DNIC- GS)	Collagen matrix/ DNIC-GS composite spongy sheets	4.0 μM DNIC-GS	Complete NO release from matrix in 1 h after submersion in distilled water	*In vivo*	Rats	DNIC on skin wound healing	Enhanced growth, maturation, and fibrous transformation of granulation tissue
Kim et al. (2015)	GSNO	CS/NO-releasing film	0.625 mM GSNO (2.5, 10 and 20 wt%) in 20 g of chitosan solution	Ongoing NO release at 48 h for all concentrations	*In vitro*	Rats	CS/NO releasing films on wound healing	Increased wound healing and epithelialization compared to chitosan only films
Choi et al. (2020)	GSNO	CS/NO-releasing film	0.26 μM NO/mg film	Continued NO release up to day 3	*In vitro*	Mice	CS/NO releasing films on diabetic wound healing	Enhanced antibacterial activity against MRSA; Greater anti-biofilm activity; Faster biofilm dispersal, wound size reduction, epithelialization rates and collagen deposition than untreated and chitosan only groups
Baldik et al. (2002)	SNO-BSA	Demineralized bone matrix	0.3 mM/L nitrosobovine serum albumin	Not reported	*In vivo*	Rats	Femoral bone healing defect recovery	Increased union, mineral density, cortex modeling
Storm et al. (2014)	NONO xerogels	Xerogel- coated glass slides	Total NO released, μM cm^−2^: 3.3 ± 0.4, 2.5 ± 0.6, 2.6 ± 0.3, 1.9 ± 0.3, 2.3 ± 0.3 (0, 6, 12, 18, 24 coating layers, respectively)	Apparent t_1/2_, h: 11.4 ± 0.7, 13.6 ± 1.4, 17.8 ± 4.3, 13.2 ± 0.6, 16.3 ± 2.4 (0, 6, 12, 18, 24 coating layers, respectively)	*In vitro*	Mice fibroblasts	NO-releasing superhydrophobic xerogel effect on Pseudomonas	Reduction in *Pseudomonas aeruginosa* compared to control
Diwan et al. (2000)	CBC-NO	Surgically implanted NO-releasing chitosan matrix	200 mg CBC-NO (releases 250 nM NO per 5 mg of CBC-NO)	Duration of NO release = 185 min	*In vivo*	Rats	NO impact in femoral fracture repair	Day 17 post-fracture: 20% increase in cross-sectional area fracture callus compared to control; 30%
								compared to NOS inhibition
Differ et al. (2019)	Deta NONOate, SNAP, L-Arginine		Deta NONOate (10–1,000 μM) SNAP (1–100 μM) Arginine (0.1–7.5 mM)	Deta NONOate t_1/2_ = 20 h SNAP t_1/2_ = 6 h	*In vitro*	C2C12BRELuc reporter cell line	BMP2- induced signaling and osteogenic abilities	Enhanced BMP2 signaling and osteogenic induction with all NO donors studied
Charville et al. (2008)	NO diazeniumdiolate-modified xerogels; PVC coated	Xerogel- coated glass slides	10, 20, 30 and 40% AHAP3 xerogels (average NO flux, pM cm^−2^ s^−1^: 11, 18, 23, 30, respectively)	Not reported	*In vitro*		Bacterial adhesion to fibrinogen coated NO releasing surfaces	Reduced bacterial adhesion for *Staphylococcus aureus, Escheria coli* and, *Staphylococcus epidermidis*
Hetrick and Schoenfisch (2007)	NO xerogels	Xerogel- coated glass slides	10, 20, 30, and 40% AHAP3 xerogels	Low-level NO release up to 16 h at 25°C	*In vitro*		Pseudomonas adhesion	NO xerogels showed inhibition of *Pseudomonas aeruginosa* and killing of adhered bacterial cells
Privett et al. (2010)	Surface generated NO using model xerogel surfaces (AHAP3 and BTMOS)	Xerogel- coated glass slides	10, 20, 30, and 40% AHAP3 xerogels (NO release over 15 h at 37°C, μM cm^−2^: 0.049 ± 0.004, 0.324 ± 0.055, 0.852 ± 0.323, 2.077 ± 0.656, respectively)	t_1/2_, h (37°C): 2.450 ± 0.272, 2.853 ± 0.231, 2.358 ± 0.274, 3.9364 ±0.381, respectively	*In vitro*		Surface generated NO against *Candida albicans* using modified xerogel surfaces	Reduction in Candida growth
Holt et al. (2011)	Diazeniumdiolate NO donor-functionalized xerogels	Surgically implanted	0.28 ± 0.11 μM cm^−2^ total NO released, 20 ± 7 pM cm^−2^ s^−1^ max NO flux	t_1/2_ = 4 h No NO release detected after 7 days	*In vivo*	Rats	Quantify incidence of bacterial infection in implanted xerogel coated titanium pins	Reduced infection incidence, decreased erythema and edema surrounding surgical wounds
Riccio et al. (2009)	Sol-gel derived silica nanoparticles: NO-releasing RSNO-modified xerogels	Incubated with fibroblasts	20, 40, 60, and 80% MPTMS xerogels (Total NO released, μM mg^−1^: 0.47 ± 0.13, 0.68 ± 0.07, 0.81 ± 0.03, 1.31 ± 0.07 for, respectively)	NO flux > 0.4 pM cm^−2^ s^−1^ for up to 3 days with 20% MPTMS gel and up to 1–2 weeks with 40–80% MPTMS gel	*In vitro*	Mouse fibroblasts	Examine ability of xerogel coatings to resist bacterial and platelet adhesion	Reduction in *Pseudomonas aeruginoasa* and activated platelet adhesion in RSNO-modified xerogels, with maintenance of fibroblast viability
Hetrick et al. (2008)	NO- releasing silica nanoparticles	Incubation	~3.8 μM·mg^−1^ total NO released, ~21,700 ppb·mg^−1^ max NO flux	t_1/2_ = 18 min	*In vitro*	Mouse fibroblasts	Examine NO-releasing silica nanoparticles bactericidal effectiveness	NO delivered from silica nanoparticles more effective at killing *P. aeruginosa*
Lu et al. (2013)	PAMAM dendrimers	Incubation	~1 μM/mg total NO release	t_1/2_ ~ 1 h	*In vitro*	Mouse fibroblasts	Evaluation of PAMAM bactericidal properties	Size and exterior functionality crucial in dendrimer-bacteria association, NO delivery efficiency, bacteria membrane disruption, migration of biofilm and mammalian toxicity
Johnson et al. (2010)	Nitrosthiol	G4-SNAP scaffold	2 μM SNAP with 0.5–10 mM GSH, 1.28 μM NO/mg max NO release	Not reported	*In vitro*	Rat Heart (isolated, perfused)	Evaluation of G4-SNAP for reducing ischemia-reperfusion injury)	Dendrimer scaffold did not inhibit NO therapeutic activity
Lin et al. (2018)	*In situ* self-assembling NO-releasing micelle deposits	Subcutaneously implantation	30 μM NONOate	t_1/2_ = 1298.3 ± 205.8 s w/ Hb at 37°C	*In vivo*	Ovariectomized rats with osteoporosis	Examine ability of self-assembling micelles to release NO	Longer NO-released in OVX-induced osteoporosis rats reversing effects
Duong et al. (2014)	CCS Polymers	Incubation	60 μM total NO release	Continuous NO release for 70 h	*In vitro*		Evaluate CCS controlled NO release	Decreased cell attachment and biofilm production of *Pseudomonas aeruginosa* with CCS polymers
Pant et al. (2019)	SNAP	3D bone scaffolds	10 wt% SNAP, initial NO release rate 0.5 ± 0.06 ×10^−10^ mol/min/mg, NO release rate 0.23 ± 0.02 ×10^−10^ mol/min/mg after 24 h	Theoretical t_1/2_ extrapolated to 8.6 days	*In vivo*	Mice fibroblasts	Examination of 3D bone scaffold releasing SNAP anti-bacterial properties	Reduction in *Staphylococcus aureus* and *Pseudomonas aeruginosa* adherence
Friedman et al. (2011)	Sol-gel derived silica nanoparticles: NO-np generating GSNO	Incubation	~300 μM GSNO release	Duration of NO release >24 h	*In vitro*	Human clinical isolates	Examine NO-np GSNO generating abilities	NO-np are able to generate and maintain GSNO formation over prolonged time period, where lower NO concentrations are more effective antimicrobial agents

## No Donor Molecules

**Figure 5:**
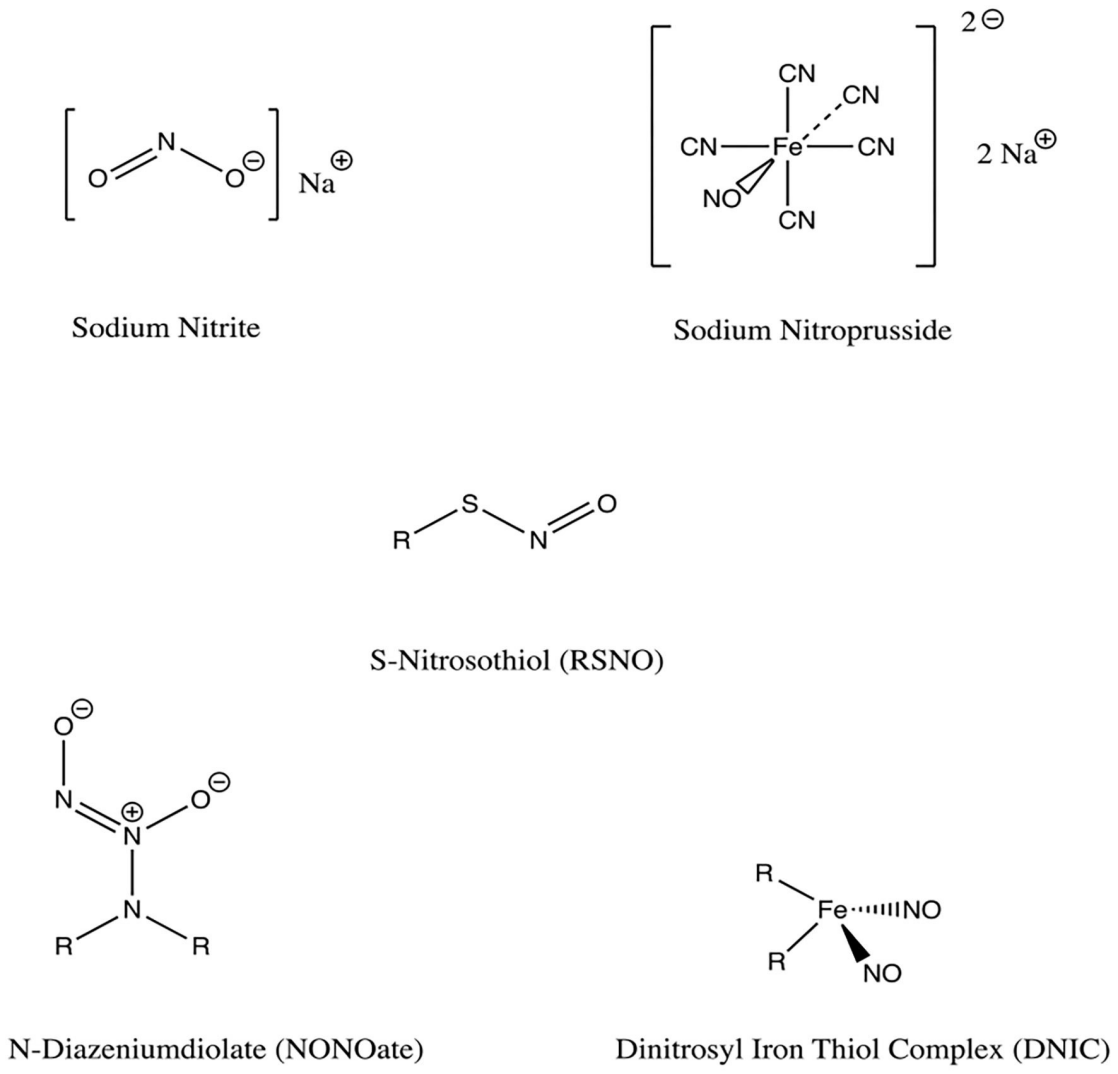
Chemical structures of NO donors studied in bone and tissue healing. There are a variety of NO donor molecules that store and release NO, independent of the enzymatic actions of NOS.

NO donors are functional groups that store and release NO, independent of the enzymatic actions of NOS (Figure 5). There is a wide array of NO-releasing materials which have emerged as potential therapeutic options for a spectrum of pathologies including cancer, bacterial infection, wound healing applications, and cardiovascular disease (Carpenter and Schoenfisch, 2012). While NO can be delivered systemically using hyperbaric therapy, this approach has been limited in practice by the need for continuous oversight of the patient during treatment and the hazards of pressurized NO gas cylinders (Malone-Povolny et al., 2019). This has prompted investigation of NO donors as local delivery vehicles for the enhancement of fracture and wound healing; among these, *N-*diazeniumdiolates (NONOates) and *S-*nitrosothiols (RSNOs) are the most prominent and diverse (Nichols et al., 2012; Malone-Povolny et al., 2019). Other classes include organic nitrites, metal-NO complexes, and nanoparticulated delivery vehicles. Depending on their formulation, NO donors may either require enzymatic catalysis, or release NO spontaneously, and may result in a variety of metabolites which should be considered (Pant et al., 2019). Varying release kinetics results in different concentrations and durations of NO delivery (Pant et al., 2019), another important consideration as low and high levels of NO lead primarily to activation of osteoblasts and osteoclasts, respectively (Wimalawansa, 2010).

### Organic Nitrates/Nitrites

Organic nitrates exist as esters between mono/polyhydric alcohols and nitric acid. Similarly, organic nitrites exist as esters between alcohols and nitrous acid. These compounds can be synthesized either by alcohol esterification using nitric acid, by a reaction of silver nitrate with alkyl halides, or by reacting alcohols with nitrous or other nitrosating agents (Wang et al., 2002; Yang et al., 2018). The release of NO from these compounds occurs without the need for exogenous enzymes, as nitrates are bioactivated by mitochondrial aldehyde dehydrogenase (Nichols et al., 2012).

In practice, NO can be generated from these organic compounds by combining inorganic sodium nitrite cream with a cream made of either citric or ascorbic acid. This method can be easily accomplished by applying the two creams to a wound site, and has been shown to enhance re-epithelialization and wound closure rates in rats and mice (Zhu et al., 2007, 2008). In tissues, nitrite itself has also been shown to serve as a source of NO through conversion of nitrite to NO via xanthine oxidase, particularly in acidic or hypoxic settings (Li et al., 2008). The application of acidified nitrite creams has also been studied in randomized controlled trials on human wounds with promising results, both in the setting of non-infected ulcers (Phillips et al., 2004) and in eradicating MRSA from infected wounds (Ormerod et al., 2011). NO exhibits a dose-dependent ability to eradicate MRSA, a common culprit for biofilms that plague fracture-associated infections (Ormerod et al., 2011). Limitations of this therapy include the cessation of wound healing benefits when NO is withdrawn, potential for mucosal irritation, and the uncertainty with regard to the optimum frequency and duration of therapy.

As an alternative to acidified nitrite suspended in ointment, Friedman et al. constructed a nitrite-containing hydrogel/glass composite nanoparticle system that released NO in a controlled, sustained manner (Friedman et al., 2008). This system involved the thermal reduction of nitrite to NO within glassy matrices comprised of tetramethylorthosilicate, polyethylene glycol, and chitosan. The NO is then released from this matrix by exposure to moisture over extended periods of time. This hydrogel/glass composite produced a steady state concentration of NO that was achieved and maintained for at least 24 h, compared to a control sample of dissolved gaseous NO which rapidly peaked and returned to baseline levels within 5 min (Friedman et al., 2008). Wound closure was found to be accelerated with this NO delivery system against *MRSA* and *Acinetobacter baumannii* mice (Martinez et al., 2009; Mihu et al., 2010).

### Metal-NO Complexes

Metal-NO complexes, or metal nitrosyls, are NO ligand coordination complexes (Yang et al., 2018). Iron is the most widely used metal, as in sodium nitroprusside for example. Iron-sulfur complexes have been used, as in Roussin's black salt (RBS), red salt (RRS) and red esters (RREs). To synthesize these metal-NO complexes, nitrosyls and sulfide salts or thiols are reacted, and light is commonly used as a trigger for stimulating release of NO from the metal complexes (Yang et al., 2018). Metal nitrosyls can release NO enzymatically or non-enzymatically, in the presence of vascular tissue, reducing agents, or light (Nichols et al., 2012).

Metal-NO complexes have wound-healing potential, though they currently are not applied clinically for this purpose. The main clinical use of sodium nitroprusside (SNP) is the reduction of blood pressure by vasodilation in hypertensive emergency, however SNP can also release NO in the body by both enzymatic and non-enzymatic reactions (Wang et al., 2002). In rat osteoblast-enriched cell cultures, SNP-mediated release of high NO concentrations inhibits cell proliferation and induces apoptosis, with no effect on alkaline phosphatase (ALP) (Mancini et al., 2000). This mechanism is similar to the high-concentration NO activity seen in the pro-inflammatory response following iNOS activation (Mancini et al., 2000). Another study concluded that low concentrations of SNP might be useful in promoting bone repair by increasing matrix deposition and ALP activity (Abnosi and Pari, 2019).

Dinitrosyl Iron Thiol Complexes (DNICs), formed by iron and NO, have shown promise in soft tissue healing in rodent studies. When applied directly to the wound, DNICs enhanced angiogenesis and wound healing in a diabetes model (Chen et al., 2019) and full thickness skin wound model (Shekhter et al., 2015; Chen et al., 2019). In particular, DNIC-1 [Fe_2_(μ-SCH_2_CH_2_OH)_2_(NO)_4_] was shown to exhibit sustained NO release with a half-life of 27.4 h at 25°C (Chen et al., 2019). Notably, DNIC is present within bone marrow-derived macrophages and released by cytotoxic activated macrophages (Vanin et al., 1993), though it remains to be elucidated how the mechanism of macrophage induced DNIC/NO release can be applied to optimizing fracture healing. Despite the potential benefits of metal-NO compounds, there is concern for cellular toxicity due to release of cyanide and formation of peroxynitrite, which is cytotoxic (Vanin et al., 1993).

### Low Molecular Weight NO Donors

The majority of NO-donor treatments use N-diazeniumdiolates (NONOates) or S-nitrosothiols (RSNOs) (Nichols et al., 2012; Malone-Povolny et al., 2019). Both are capable of releasing NO spontaneously in physiologic media without requiring other agents. They can be used both in nano-particle formulations as well as the non-nano formulations discussed here. The primary limitations in employing these molecules for clinical use are uncontrolled NO release and the formation of cytotoxic precursors. Encapsulation of these NO donors within scaffold polymers or attachment by covalent binding to a scaffold structure can be used to combat these issues (Malone-Povolny et al., 2019). Albumin and chitosan have been synthesized and studied as scaffolds for drug delivery of RSNOs and NONOates (Riccio and Schoenfisch, 2012).

RSNOs are endogenous carriers of NO that can be synthesized on free thiol groups through exposure to a nitrosating agent (Malone-Povolny et al., 2019). RSNO degradation is photosensitive, especially to UV light, and degradation can also be catalyzed by copper ion and ascorbate (Wang et al., 2002). RSNOs are unstable at room temperature, a property that can be partially overcome by the addition of alkyl groups to form tertiary RSNOs (Wang et al., 2002). Physiologically, photodermal decomposition is the most prominent mechanism of NO release (Malone-Povolny et al., 2019). Storage of dry samples in dark and cold environments can prevent instability, however it may also decrease viability in clinical settings (Malone-Povolny et al., 2019). The release kinetics of RSNOs may be modified by manipulation of structural and environmental conditions as well as incorporation into drug delivery scaffolds.

Several subtypes of RSNOs have demonstrated enhanced wound and fracture healing *in vivo*. S-nitrosoglutathione (GSNO) is one such subtype and serves as both a free NO donor and also as an S-nitrosylating agent of protein thiols through a process called transnitrosation, resulting in increased tissue nitrosylation and protection from oxidative stress (Sun et al., 2006; Broniowska et al., 2013). Kim et al. prepared GSNO on a chitosan film dressing for use on full-thickness wounds, and showed sustained, sufficient NO release to the wound bed, dose-dependent antibacterial activity against both Gram-positive and Gram-negative organisms, and accelerated epithelialization and reduction in wound size (Kim et al., 2015). A similar experiment by Choi et al. showed the GSNO-chitosan film significantly enhanced anti-biofilm activity, in addition to promoting wound healing (Choi et al., 2020). In an *in vivo* rat bone defect model, GSNO application was found to significantly enhance bone healing by enhancing the stability of the fracture hematoma architecture (Wang et al., 2016). Another RSNO subtype, S-nitrosobovine serum albumin (SNO-BSA), has been used with demineralized bone matrix as a bone graft material in a rat femoral defect model (Baldik et al., 2002). This study demonstrated a 62% increase in union across boney defects in rats treated with SNO-BSA compared to control, in addition to mineral density augmentation and cortex modeling (Baldik et al., 2002).

**Figure 6:**
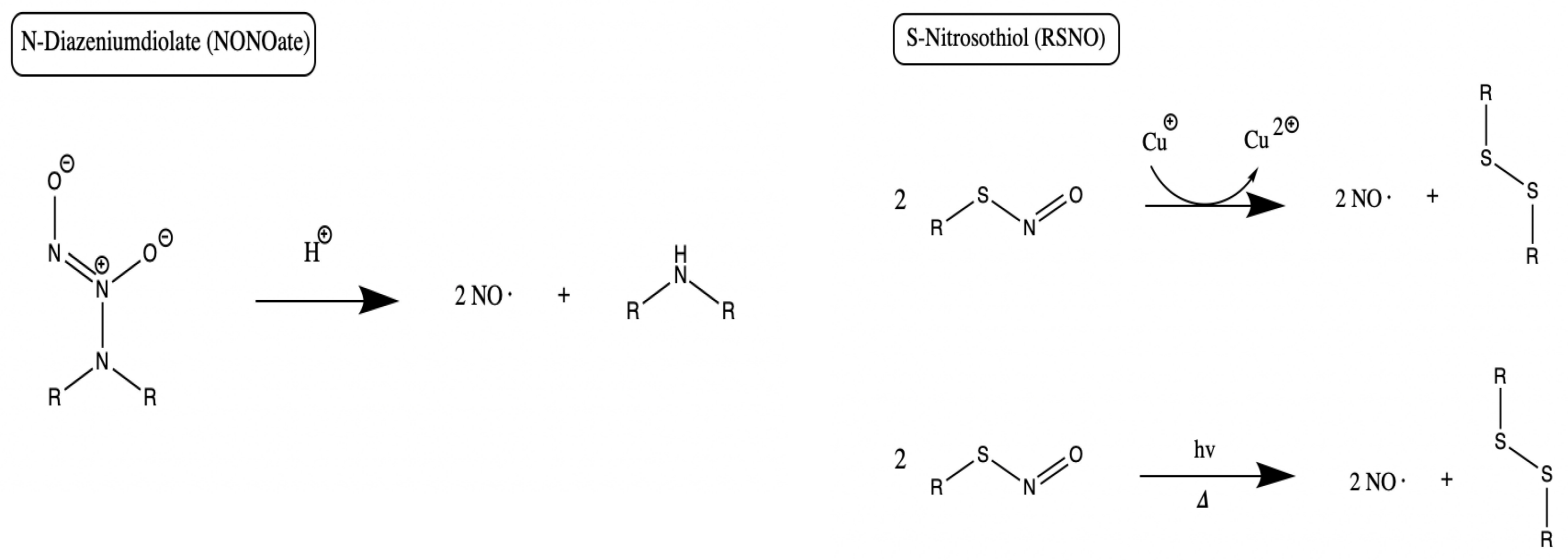
NO release mechanisms from NONOates and RSNOs. Primary release mechanisms of two major classes of NO donors, as described by Schmidt et al. (1997) and Nichols et al. (2012). Of note, in addition to releasing NO, RSNOs can also act through S-nitrosylation of protein thiols in tissues, a process called transnitrosation (Broniowska et al., 2013).

NONOates are a group of compounds having two nitrogen atoms and two alkyl groups, and they also demonstrate the capacity to serve as NO donors (Homer and Wanstall, 1998) (Figure 6). The compounds form on secondary amines under high gaseous NO pressure in alkaline solution, and require storage in an anhydrous environment (Malone-Povolny et al., 2019). Most are bound to nitrogen or carbon at the NO group, though oxygen and sulfur bound NONOates also exist (Yang et al., 2018). At physiologic pH in aqueous media, NO release occurs by spontaneous proton-initiated hydrolyzation. Temperature, local pH, and the structure of the NONOate can be modified to influence NO-release kinetics (Malone-Povolny et al., 2019). Low molecular weight NONOates and NONOate-modified macromolecular scaffolds have both been utilized for NO delivery in wound and bone healing (Malone-Povolny et al., 2019). They have been used in fracture and bone healing, as well as prevention of post-operative infections (Nichols et al., 2012). A class of superhydrophobic materials have been created with NONOates and examined in their antimicrobial function against *Pseudomonas aeruginosa* (Storm et al., 2014). Spray-coated with fluorinated silane/silica composite, superhydrophobic NO-releasing xerogels were applied to NONOate modified xerogels and demonstrated reduced viable bacteria compared to control in murine fibroblasts (Storm et al., 2014).

In a study to evaluate the effect of local NO administration on fracture healing by Diwan et al., a rat femoral fracture model was evaluated after the implantation of a NONOate derivative of carboxybutyl or chitosan, with or without systemic administration of a NOS inhibitor. Rats with the NONOate implant exhibited a 20% increase in callus cross-sectional area compared to control, and a 30% increase compared to NOS inhibition group (Diwan et al., 2000). In another study, direct local NO application with both a NONOate and RSNO derivative *S-*nitroso-*N-*acetyl-_DL_-penicillamine (SNAP) was found to enhance BMP2-mediated osteogenic activity (Differ et al., 2019). These results support the potential for use of NONOates in bone filling, such as synthetic grafts with bone promoting factors like BMP (Bishop and Einhorn, 2007).

## No Donor Nanomaterials

Nanomaterials are the current subject of much investigation as NO-releasing scaffolds, given emerging evidence of their effectiveness in delivering NO to aid in augmenting wound healing (Malone-Povolny et al., 2019). Nanomaterials offer a variety of benefits which can be applied to NO delivery to bone. They allow for encapsulation of desired the therapeutic compound to ensure controlled and sustained release (Singh et al., 2019). When particle size is on the order of nanometers as opposed to the typical micron-sized material used for conventional therapeutics, the overall particular surface area increases by several orders of magnitude (Singh et al., 2019). Given surface-level interactions with surroundings *in vivo*, nanomaterials exhibit significantly higher potential for biologic system interaction. Furthermore, nanomaterials can be tuned to specific parameters to ensure optimal interaction within a system. For example, Slomberg et al. investigated the potential for aseptic activity against *P. aeruginosa* and *S. aureus* biofilms in NO-releasing silica particles by modulating the aspect ratio of the silica particles (Slomberg et al., 2013). These authors varied the aspect ratio of the NO-releasing silica particles from 1 to 8 while maintaining constant particle volume (~0.02 μm^3^) and NO-release totals (~0.7 μmol/mg) (Slomberg et al., 2013). They determined that the optimal configuration of silica Nitric oxide-releasing particles with regards to activity against bacterial biofilms was decreased size but increased aspect ratio (Slomberg et al., 2013). The potential for nanomaterial tuning is an additional benefit of the utilization of such material as potential NO donors.

To date, nanomaterial donors of NO have not been utilized as extensively for bone healing and fracture repair, for reasons ranging from complicated synthesis to thermodynamic stability (Seabra and Duran, 2017). These challenges may be partially mitigated by using nanomaterial scaffolds to encapsulate NO donors in a hydrophobic microenvironment (Seabra and Duran, 2017), a strategy which also reduces nanomaterial toxicity and tendency to be phagocytosed (Malone-Povolny et al., 2019). We aim to highlight the existing evidence surrounding nanomaterial delivery of NO to aid in fracture and bone healing, as well as decrease biofilm formation. These nanomaterials include NO-releasing xerogel polymer composites, sol-gel nanomaterials, dendrimers, micelles, core cross-linked star polymers, and polymeric 3D NO-releasing scaffolds.

### NO-Releasing Xerogel Polymer Nanocomposites

Avoidance of infection and biofilm formation is essential for fracture healing to occur (Thomas and Puleo, 2011). The antimicrobial and antibiofilm capabilities of NO have inspired the development of nanoNO-releasing xerogel polymers for inhibition of bacterial and fungal adhesion. NO-releasing xerogel polymer composites (aminopropyl trimethoxysilane (AHAP3) and isobutyltrimethoxysilane (BTMOS), Mercaptosilane-modified, and *S-*nitrosothiol-modified xerogels) have been shown to reduce bony adhesion of bacterial and fungal organisms including *Pseudomonas aeruginosa, Staphylococcus aureus, Staphylococcus epidermidis, Candida albicans*, and *Escherichia coli* (Charville et al., 2008). Additionally, xerogels have been shown to reduce bacterial adhesion to fibrinogen-coated surfaces (Hetrick and Schoenfisch, 2007; Charville et al., 2008; Privett et al., 2010). Clinically, this broad spectrum of antimicrobial action could assist in eradicating common fracture infections, and enhance treatment of multi-drug resistant organisms (Nablo et al., 2005). Similarly, NO-releasing xerogel-coated external fixation pins have been studied in rats, showing lower incidence of infection supporting the potential of NO-releasing xerogels in reducing infection even in multi-drug resistance bacteria like *Staphylococcus auerus* (Holt et al., 2011). Given that external fixators are often used in the setting of contaminated open fractures, these NO-releasing xerogel polymer composites could potentially be of significant clinical value. The rate of NO release from NONOate-modified xerogels varies based on the type of aminosilane precursor molecule, with NO release half-lives ranging from 1.7 to 5.7 h (Storm and Schoenfisch, 2013). However, the most important factor influencing NO release rates has been shown to be the hydrophobicity of the xerogel matrix, rather than intramolecular NONOate stabilization by 1° amines (Storm and Schoenfisch, 2013). Riccio et al. used sol-gel chemistry to form RSNO-modified xerogels as NO-releasing coatings, and sought to explore the ability of the coating to resist bacterial and platelet adhesion (Riccio et al., 2009). This composite allows water channels inside the particles to open, permitting the release of NO over prolonged periods of time (Friedman et al., 2011). In addition to the work by Riccio et al., Sol-gel derived delivery systems can also incorporate nanomaterials for the delivery of NO.

### NO Delivery From Sol-Gel Derived Nanomaterials

**Figure 7:**
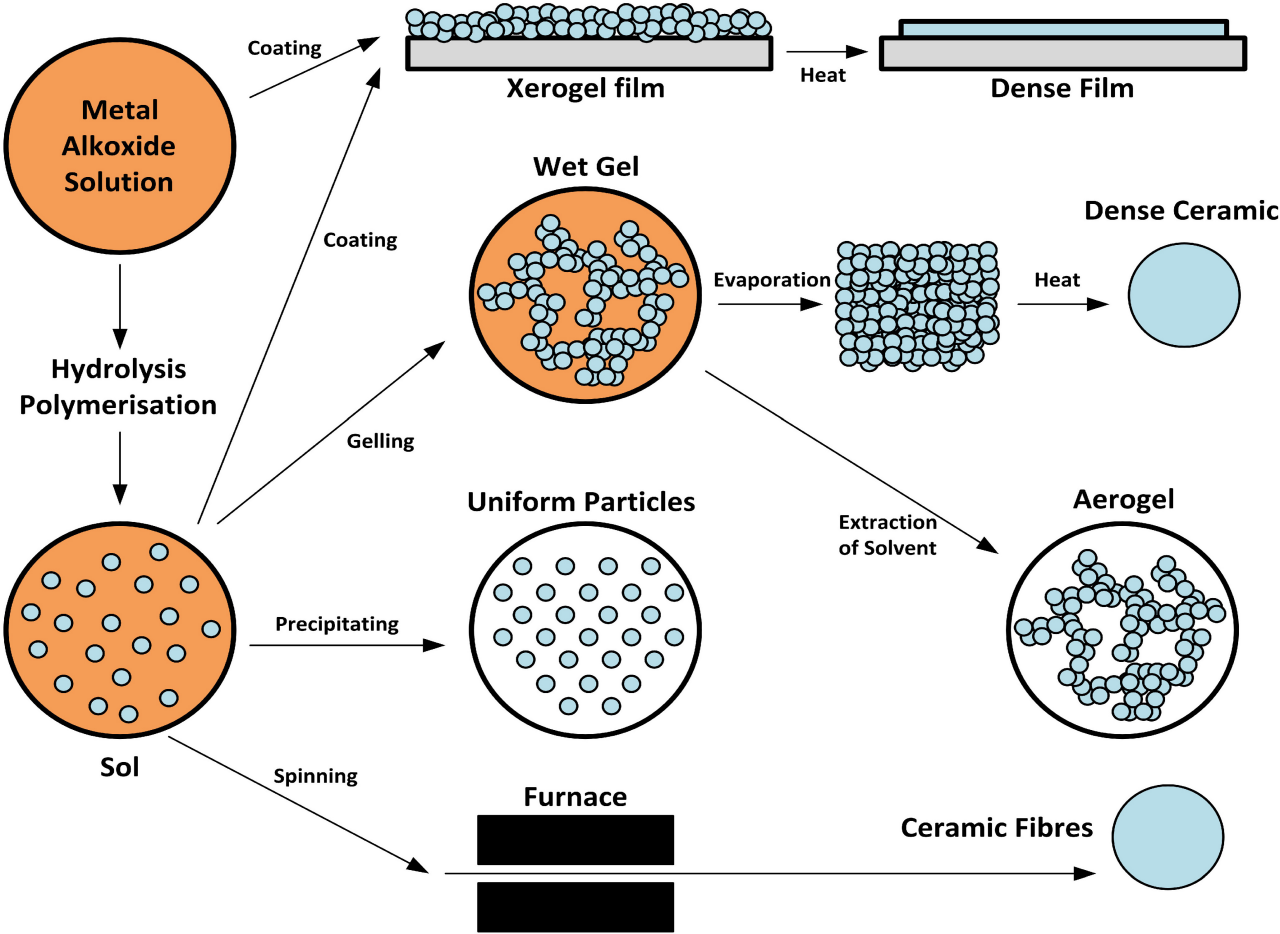
Synthesis of xerogels and sol gels. Both xerogels and sol-gels are nanomaterials that may function as NO-releasing coatings for implanted materials when NO donors are conjugated to the gel. This figure is a reproduction of an original image by Claudionico, which is licensed under a Creative Commons Attribution 3.0 Unported License (https://creativecommons.org/licenses/by-sa/3.0/legalcode); https://commons.wikimedia.org/wiki/File:Sol-Gel_Technology_Scheme.png.

Sol-gels using silica-based materials have been studied in bone disease given their uniform pore size and distribution (Czarnobaj et al., 2019) (Figure 7). Silica nanoparticles have been additionally used as RSNO scaffolds. Malone-Povolny and Schoenfisch synthesized and characterized RSNO-functionalized mesoporous silica nanoparticles (MSN). The RSNO modified MSNs were coated into polyurethane, extending NO release and increasing NO payloads (Malone-Povolny and Schoenfisch, 2019). Silica is also cost-effective, possesses a strong surface energy, and has the chemical versatility and high loading capacity necessary for drug delivery (Czarnobaj et al., 2019). Hetrick et al. used NO-releasing silica nanoparticles aimed at reducing *Pseudomonas aeruginosa* disease burden (Hetrick et al., 2008). They showed enhanced bactericidal capacity and reduced cytotoxicity to mammalian fibroblasts when compared to a NONOate NO donor (Hetrick et al., 2008). These specific NO-releasing silica nanoparticles had an NO-release half-life of 18 min, compared to the low molecular weight NO donor, PROLI/NO, which has a half-life of 1.7 min (Hetrick et al., 2008). However, half-lives for NONOate-modified silica nanoparticles overall have been shown to range from 1 to 12 h (Shin et al., 2007).

### NO Delivery From Dendrimers

Dendrimers are advantageous for their ability to store and disperse large amounts of NO (Seabra and Duran, 2017), as well as the ability to modify their exterior by attaching molecules for imaging or targeted therapeutic applications (Stasko et al., 2008). Additionally, these materials exhibit differences in cytotoxicity which can be employed clinically. These differences depend on various factors which can be altered during the engineering process such as the nature of the terminal moieties (anionic, neutral, or cationic) and the number of surface groups included (Janaszewska et al., 2019). Still, the utility of dendrimers may be limited by their complex synthesis and associated cost (Seabra and Duran, 2017). Stasko and Schoenfisch have reported successfully using dendrimer nitric oxide scaffolds with polypropyleneimine dendrimers containing N-diazeniumdiolate to spontaneously generate NO via proton-initiated diazeniumdiolate decomposition; the NO release half-life of one particular dendrimer from their study (DAB-C7-16/NO) was 77 min (Stasko and Schoenfisch, 2006). Lu et al. evaluated the antibacterial efficacy of NO-releasing dendrimers against established biofilms and found that biofilm eradication was maximized by adding an equal proportion of hydrophobic and hydrophilic exterior modifications (Lu et al., 2013). The bactericidal action of NO-releasing dendrimers against *Pseudomonas aeruginosa* biofilms was examined, as well as cytotoxicity toward mice fibroblasts to determine optimal dendrimer size and hydrophobicity (Lu et al., 2013). Subsequently, Stasko et al. synthesized generation-4 polyamidoamine (PAMAM) dendrimers with *S-*nitrosothiol exteriors, modified either with *N-*acetyl-D, L-penicillamine (NAP) or N-acetyl-L-cysteine (NACys) (Stasko et al., 2008). While NO release was dependent on the nitrosthiol structure, the ability of G4-SNAP to inhibit thrombin-mediated platelet aggregation was 62% inhibition compared to 17% for the small molecule NO donor, demonstrating the utility for NO delivery and release via dendrimers (Stasko et al., 2008). G4-SNAP was then shown to reduced ischemia, reperfusion injury in a rat heart, as the dendrimer scaffold successfully delivered NO (Johnson et al., 2010). Further work remains to examine the efficacy of dendrimers as NO-releasing agents for delivery to bone.

### Core Cross-Linked Star Polymers

**Figure 8:**
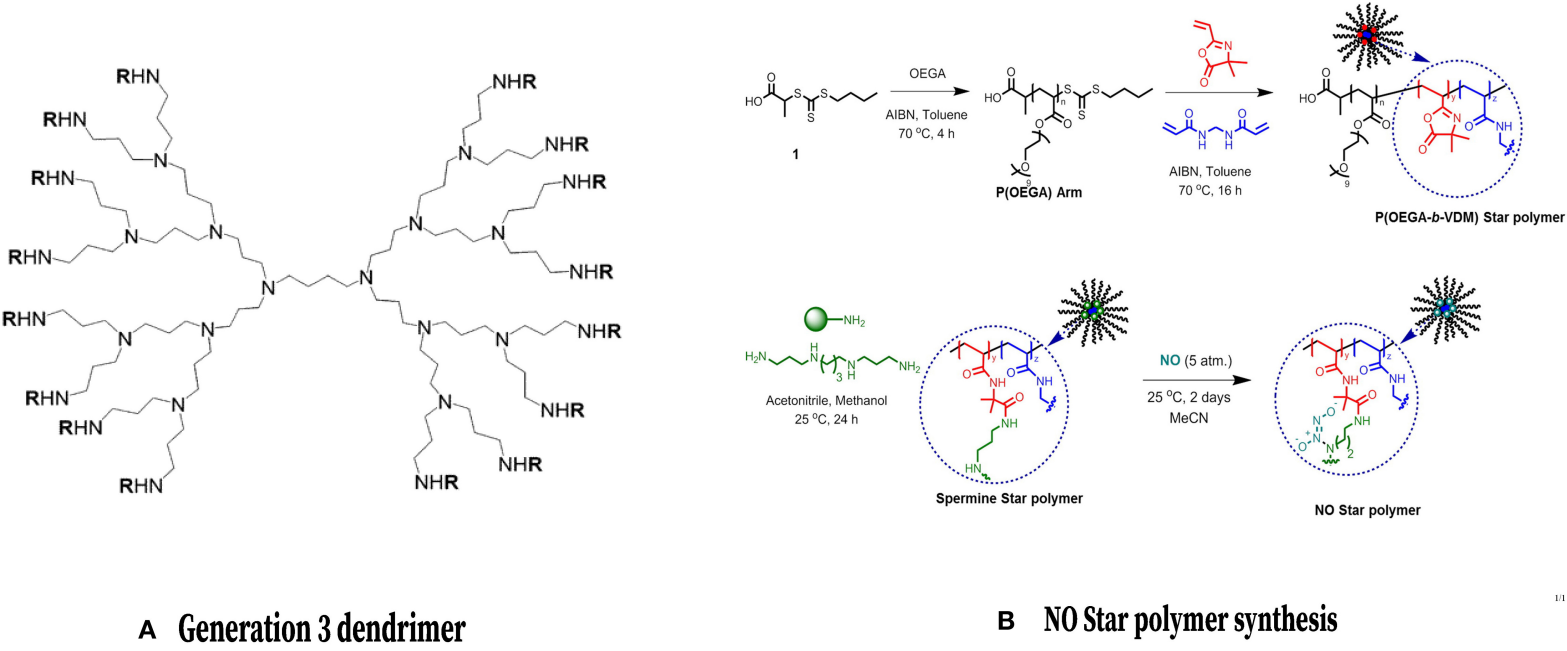
Dendrimers and Core Cross Linked Star Polymers for NO release. (Left) Chemical structure of a common NO-releasing polypropylenimine dendrimer. Reprinted (adapted) with permission from Stasko and Schoenfisch (2006). Copyright 2006 American Chemical Society. (Right) Formation of a CCS polymer and conjugation with NO. Reprinted (adapted) with permission from Duong et al. (2014). Copyright 2014 American Chemical Society.

Core cross-linked star (CCS) polymers are macromolecules made of linear polymeric arms encircling a central cross-linked core that have the ability to accumulate hydrophobic drugs within their interior as they are transported through aqueous media (Quinn et al., 2015) (Figure 8). They have been synthesized by ionic group transfer (GTP), ring-opening and controlled radical polymerizations (CRP) (Tan et al., 2010). CCS polymers can enhance NO-releasing scaffold stability and protect scaffolds from release triggers, resulting in the sustained release of NO over a period of at least 70 h, thus prolonging its anti-biofilm effect (Duong et al., 2014). This extended release period offers longer-term prevention of biofilm formation when compared to other NO donor scaffolds (Duong et al., 2014), especially in light of the fact that biofilm formation occurs over prolonged periods of time (Kostakioti et al., 2013). The major drawback of polymer therapeutics is the fast elimination from the bloodstream by the mononuclear phagocytic system (Blencowe et al., 2009).

### NO-Releasing Micelles

**Figure 9:**
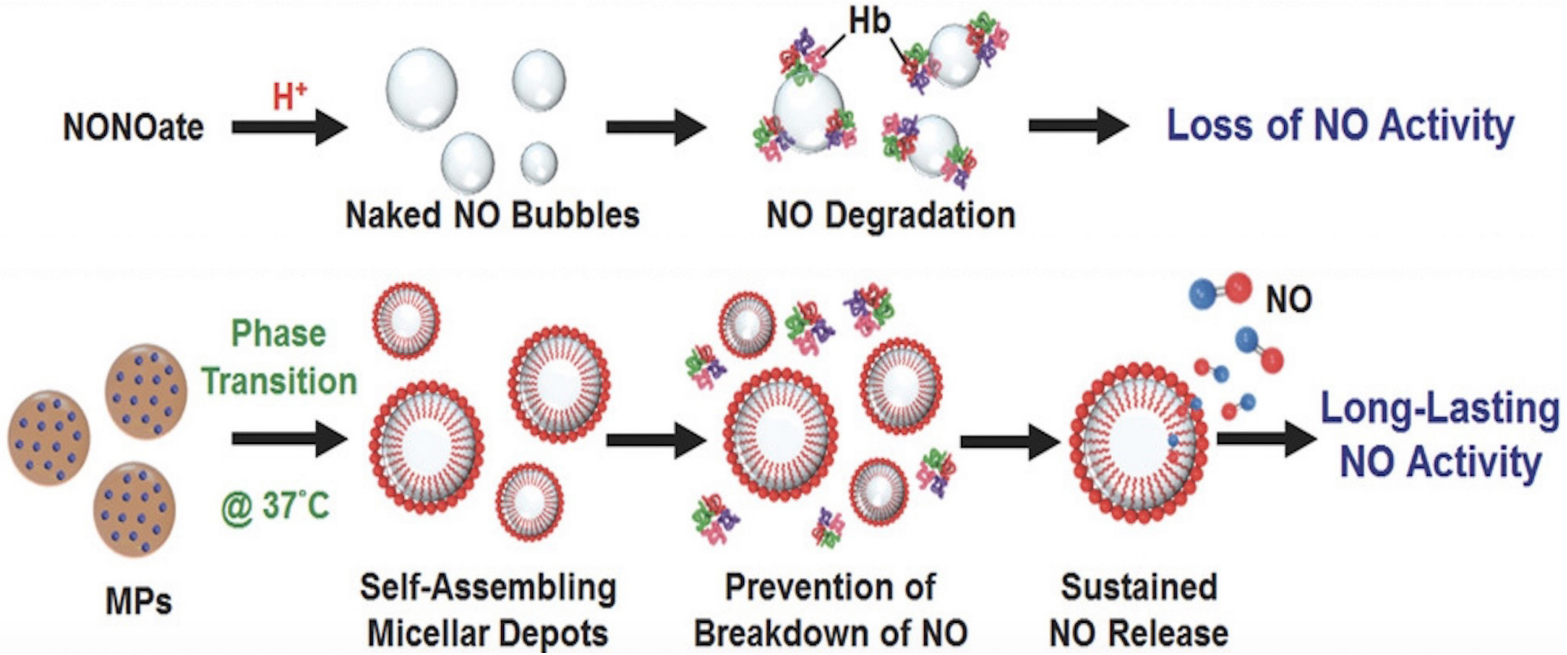
Micelles prolong release of NO to bone. Compared to NONOates which simply release free NO that can be easily degraded by hemoglobin, micelles protect NO from degradation, thus prolonging the action of NO. This figure was reprinted (adapted) with permission from the original illustration by Lin et al. (2018).

Polymeric micelles are able to store and deliver hydrophobic and hydrophilic agents, making them extremely versatile, however, their utility is limited by thermodynamic instability (Seabra and Duran, 2017). Lin et al. developed an injectable microparticle (MP) system encapsulating a NONOate donor (Lin et al., 2018). After subcutaneous injection into mice, this formulation resulted in self-assembled micelles with entrapped NO, which was released passively from the micelles yielding prolonged delivery to bone (Lin et al., 2018) (Figure 9). Specifically, the half-life of NO release generated from micelles was 21.6 min, while the half-life of NO release generated from free NONOate was only 20.6 s (Lin et al., 2018). In this osteoporosis rat model, the NO was found to inhibit bone turnover and thus produce thicker trabecular bones, denser networks and decreased fat marrow levels (Lin et al., 2018). How this technology could be used to enhance fracture healing remains to be investigated. In theory, prolonged delivery of low levels of NO could promote osteogenesis via enhanced osteoblast activity (Wimalawansa, 2010; Klein-Nulend et al., 2014; Abnosi and Pari, 2019). Additionally, NO-releasing liposomes have been developed which can be readily tuned by altering either the phospholipid composition or the NO donor molecule structure. *N*-diazeniumdiolate-encapsulated liposomal structures can enhance sustained release from the liposomes for up to 48 h. Furthermore, phospholipid headgroup surface area can serve to control NO-release kinetics by altering cellular water uptake and resultant *N*-diazeniumdiolate NO donor breakdown to freely usable NO (Suchyta and Schoenfisch, 2017).

### 3D Bone Scaffolds for Nanoparticulated NO Release

**Figure 10:**
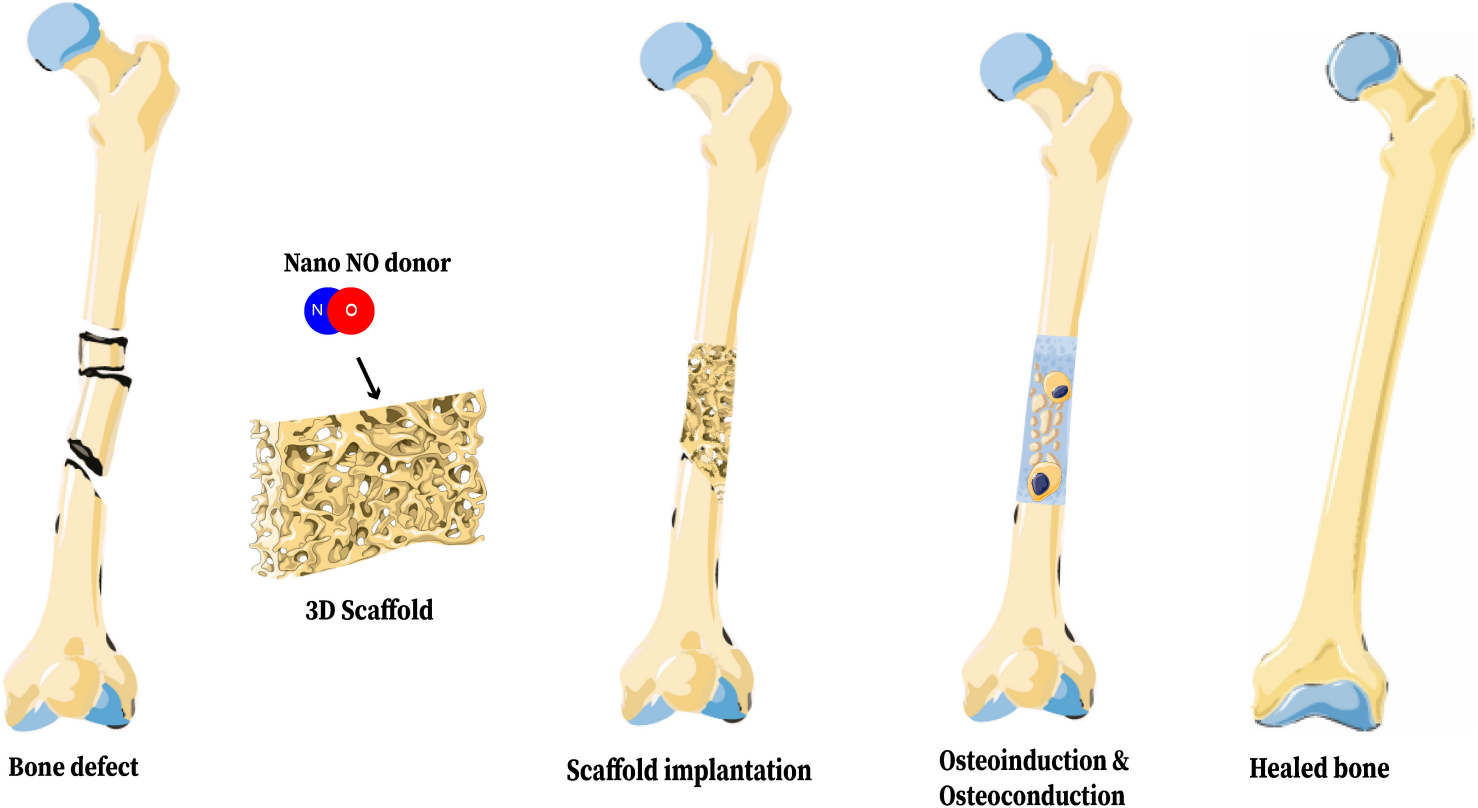
Representation of a 3D Bone Scaffold releasing nano-NO. The biologic benefits of NO on bone healing can be realized through incorporation of NO into a 3D bone scaffold and implantation of that scaffold into a fracture site. This figure was created using Servier Medical Art templates, which are licensed under a Creative Commons Attribution 3.0 Unported License (https://creativecommons.org/licenses/by-sa/3.0/legalcode); https://smart.servier.com.

Reconstructing bone has generated interest within the field of tissue engineering due to its complexity and the potential impacts of such technology. Critical-sized bone defects require reconstruction to heal, and irregular fractures or smaller segmental fractures may also require additional interventions for proper healing, partially depending on their soft tissue environment (Nauth et al., 2018). Bone is a nanocomposite of organic extracellular matrix and inorganic ceramic nanomaterials, organized in a hierarchical structure which imparts unique mechanical properties to the tissue (Alves Cardoso et al., 2012). The inorganic crystallites of bone range from 2 to 10 nm thick, 15 to 30 nm wide, and 30 to 50 nm long (Alves Cardoso et al., 2012). Thus, nanotechnology is suited to closely mimic the natural structure of bone. 3D bone scaffolds constructed from nanomaterials can maximize the mechanical strength, osteoinduction, osteoconduction, and osteointegration in fracture sites (Vieira et al., 2017). Additionally, scaffolds can be modified to contain additional nanomaterials for non-invasive *in vivo* labeling or controlled drug delivery (Vieira et al., 2017), including local NO donors (Figure 10).

Hydroxyapatite is a natural crystallite component of bone that can provide mechanical strength to 3D scaffolds as well as contribute chemical properties which promote tissue regeneration (Dan et al., 2016). Pant et al. created a scaffold from a nano-hydroxyapatite-starch-alginate biodegradable polymer, which was then loaded with the NO donor SNAP. The scaffold not only demonstrated excellent compressive strength, but also significant eradication of both *Staphylococcus aureus* and *Pseudomonas aeruginosa* (Pant et al., 2019). Furthermore, studies in mouse fibroblast cells showed no toxicity (Pant et al., 2019). The initial release rate of NO from these bone scaffolds was 0.5E-10 mol/min/mg, the release rate after 24 h at 37°C was 0.2E-10 mol/min/mg, and the total duration of NO release by the scaffolds was estimated to be 8.6 days (Pant et al., 2019). An additional NO-releasing scaffold which shows much promise in animal models is NO-releasing chitosan derivative (CBC-NONOate) (Diwan et al., 2000). Chitosan is already widely used in drug-delivery and tissue engineering, and can be functionalized with amine moieties to allow for straightforward NO storage via a pathway of diazeniumdiolate formation and subsequent NO release (Madihally and Matthew, 1999). In a rat fracture model, 200 mg of chitosan alone or CBC-NONOate was implanted into the adjacent bone tissue surrounding the fracture site. The delivery rate of the CBC-NONOate was 10 μmol of NO over a 3 h period. After a waiting period to allow for boney ingrowth, the cross-sectional area of the associated fracture callus was roughly 20% larger in the CBC-NONOate than in the group with CBC alone.

In recent years, electrospun polyurethane fibers have emerged as a potential macromolecular scaffold for NO delivery with excellent biomechanical properties. These scaffolds are created through the use of electric force to draw charged threads of a polymer solution to weave a mesh which can incorporate NO donor nanomaterials (Sun et al., 2019), such a NO-releasing silica particles. Koh et al. varied electrospun fiber diameter (119–614 nm) and mechanical strength (1.7–34.5 MPa of modulus) by altering polyurethane concentration and type (Koh et al., 2013). They were able to achieve a scaffold with ~83% porosity. Additionally, these authors modulated the NO-releasing particle composition, concentration, and size to create a variety of scaffold-donor complexes exhibiting a wide range of NO release totals and durations (7.5 nmol mg^−1^–0.12 μmol mg^−1^) and 7 h to 2 weeks, respectively (Koh et al., 2013). As a final example of a potential bone scaffold option for nanoparticulated NO release, much work is currently being undertaken in the realm of 3D printing for scaffold-based tissue engineering. These methods can be applied to deposit cells and biomaterials in a 3D matrix which can be then utilized in a variety of therapeutic settings (O'Brien et al., 2015). Through the potential to achieve precise control over the internal architecture and outer shape of the scaffold, complex structures can be fabricated artificially in a manner that closely reflects innate tissue architecture (O'Brien et al., 2015).

As an additional benefit of bone scaffold usage for nanoparticulated NO release, these 3D NO-releasing scaffolds hold potential as an antibacterial material for repairing critical-size and irregular bone defects and may be able to optimize NO's regulatory role in fracture healing via angiogenesis and osteogenic differentiation (Damoulis et al., 2007; Pant et al., 2019). *In vivo* studies are necessary for further evaluation of nano-NO-releasing bone scaffolds.

## NO Release Kinetics and Biological Impact

The half-life of free NO *in vivo* is on the order of seconds, due to its tendency to react with heme proteins such as hemoglobin. In order to extend the effective half-life of NO, a combined strategy of prolonging NO release and protecting NO donors from degradation has been employed. All NO donor nanomaterials described above serve this purpose, and thus all serve to prolong the action of NO. Through the incorporation of NO into donor nanomaterials, the release half-life of NO has now been increased to be on the order of hours instead of seconds (Stasko and Schoenfisch, 2006; Shin et al., 2007; Hetrick et al., 2008; Storm and Schoenfisch, 2013; Duong et al., 2014; Lin et al., 2018; Pant et al., 2019). This prolonged duration of action provided by nanomaterial delivery of NO is biologically beneficial, as the effect of NO on osteoblast and osteoclast function is dependent on prolonged exposure of these cells to low doses of NO (van't Hof and Ralston, 2001; Kalyanaraman et al., 2018), and the bactericidal effect of NO is also dependent on prolonged exposure of bacteria to NO (Kostakioti et al., 2013; Duong et al., 2014). The rate of NO release must be controlled and not too rapid, as rapid release of NO leads to high local doses which are cytotoxic (Klein-Nulend et al., 2014; Kalyanaraman et al., 2018; Radi, 2018). Lastly, it is important to note that in order to be able to meaningfully compare results across studies, future investigators should report NO release data in a standardized manner, being sure to include the following: normalized NO storage, NO-release kinetics (NO flux and half-life), NO payload, and therapeutic dose (the amount of NO necessary to induce bactericidal or therapeutic effects) (Yang et al., 2018).

## Conclusion and Future Directions

In this review, we have sought to highlight NO as a molecule of interest in the pursuit to optimize fracture healing. Its roles in fracture-site decontamination, mediating inflammation, and promoting angiogenesis and bone tissue remodeling could allow various points of intervention within the fracture healing cascade. The short half-life and diffusion distances of NO hold potential for targeted, local delivery to fractures. Additionally, NO's antimicrobial effects and ability to promote skin and soft tissue healing may be beneficial for complicated fractures, such as contaminated open fractures and those with implant-associated infection. Its efficacy in eradicating multidrug resistant infections make NO a potential alternative therapy to the toxic last-resort antibiotics, and an adjunct therapy for fighting biofilms. In addition to fracture healing in the orthopedic context, NO can also be considered in general surgery for cutaneous and soft tissue wound healing and enhancing vascular function.

Non-nanomaterial NO delivery has shown promise in reducing infections and increasing bone mineral density and rates of union. Nanomaterial NO delivery has primarily focused on mitigating infection that plagues fractures, though some evidence of enhanced osteogenesis has also been demonstrated. Nanomaterial NO delivery in the form of implant coatings and biodegradable scaffolds could be an area of advancement in fracture treatment. Much work remains to be carried out, both *in vitro* and in small and large animal models before these therapies can be considered for clinical trials. The timing and dosage of localized NO delivery to bone are major areas requiring further investigation in order to translate these therapies to humans. The toxicity NO released from nanomaterials and the nanomaterials themselves remain to be elucidated *in vivo*. We hope this review has provided insight into the potential applications of NO in enhancing fracture healing and that it inspires further work that may improve therapies for fracture patients.

## Author Contributions

All authors listed have made a substantial, direct and intellectual contribution to the work, and approved it for publication.

## Conflict of Interest

The authors declare that the research was conducted in the absence of any commercial or financial relationships that could be construed as a potential conflict of interest.
